# The cyclin dependent kinase (CDK)7 inhibitor BS-181 inhibits pathogenic Cryptococcus species, causing G_2_/M arrest and a splicing defect

**DOI:** 10.1080/21505594.2026.2629100

**Published:** 2026-02-17

**Authors:** Pooja Sethiya, Desmarini Desmarini, Bethany Bowring, Hue Dinh, Amy K. Cain, Chirag Parsania, Catriona L. Halliday, Sharon C-A Chen, Kim Hewitt, Julianne Teresa Djordjevic

**Affiliations:** a Centre for Infectious Diseases and Microbiology, Westmead Institute for Medical Research, Westmead, NSW , Australia; b Sydney Institute for Infectious Diseases, Faculty of Medicine and Health, University of Sydney, Camperdown, NSW , Australia; c ARC Centre of Excellence in Synthetic Biology, School of Natural Sciences, Macquarie University, North Ryde, NSW, Australia; d Cancer & Gene Regulation Laboratory Centenary Institute, The University of Sydney, Camperdown, NSW, Australia; e Centre for Rare Diseases & Gene Therapy Centenary Institute, The University of Sydney, Camperdown, NSW, Australia; f School of Medical Sciences, Faculty of Medicine & Health, The University of Sydney, Camperdown, NSW, Australia; g Centre for Infectious Diseases and Microbiology Laboratory Services, Institute for Clinical Pathology and Medical Research, New South Wales Health Pathology, Westmead Hospital, Westmead, NSW; h Department of Infectious Diseases, Westmead Hospital, Westmead, NSW, Australia; i Westmead Bioresources Facility and Scientific Platforms, Westmead Institute for Medical Research, Westmead, NSW, Australia

**Keywords:** *Cryptococcus neoformans*, *Cryptococcus gattii*, CDK7 inhibitor, BS-181, cell cycle, transcription, translation, splicing, antifungal

## Abstract

The fungal priority pathogen and basidiomycete, *Cryptococcus neoformans (Cn)*, causes lung and brain infection in predominantly immuno-compromised individuals and there is an urgent need for new treatment options. The pyrazolopyrimidine-based cyclin dependent kinase (CDK)7 inhibitor, BS-181, has anticancer properties, but its antifungal activity has not been investigated. We show that cryptococcal CDK7 more closely resembles the human enzyme than that of ascomycetes, and that BS-181 inhibits its activity. BS-181 inhibited growth of both *Cn* and *Cryptococcus gattii* (*Cg*), but not ascomycete fungi and delayed progression through the G_2_/M phase of the cell cycle. Transcriptomic analysis revealed that BS-181 induces splicing defects leading to elevated intron retention within the transcriptome and also suppresses translational processes. BS-181 displayed additive or synergistic activity with licensed antifungals against laboratory and clinical *Cn* and *Cg* strains, most notably with amphotericin B where synergy (2–4-fold reduction in the amphotericin B MIC) was achieved using low-sub micromolar concentrations of BS-181. Compared with either drug alone, BS-181-AmB combination therapy provided greater protection against *Cn* infection in a wax moth model (*p* ≤ 0.032) and extended survival of *Cn*-infected mice. These findings demonstrate that CDK7 inhibitors, already of interest as anticancer agents, could be repurposed to prevent or treat opportunistic fungal infections in cancer patients when combined with licensed antifungals limited by either toxicity or resistance.

## Introduction

CDK7 is an essential kinase in all eukaryotes. In mammalian cells, it regulates both the cell cycle and transcription by phosphorylating cell-cycle associated and transcriptional CDKs on their T-loop [1,2]. CDK7 is inactive as a monomer [3]. When bound to cyclin H, its substrate specificity is restricted to cell cycle-associated CDKs [3]. In contrast, when associated with Cyclin-H and the Ring Finger Assembly factor Mat1 to form the trimeric CDK Activating Kinase (CAK) complex, substrate specificity extends to transcriptional CDKs [3]. Recruitment of the general transcription factor TFIIH enables phosphorylation of the largest subunit of RNA Polymerase II (RNAPII), Rpb1, on Ser5 of its C-terminal domain (CTD), initiating transcription [3,4]. CDK7 can also phosphorylate the transcriptional CDK, CDK9 [5], which promotes CDK9-mediated phosphorylation of Ser2 of the Rpb1 CTD, an essential modification for transcription elongation [2,6]. Expression of CDK7 is upregulated in numerous human cancers, where transcription and the cell cycle are often dysregulated [7]. Cancer cells thereby become dependent on transcription, providing an Achilles heel for treatment intervention. CDK7 inhibitors are therefore promising chemotherapeutic options, and several have entered clinical trials to treat a variety of cancers [8–10].

In contrast to human cells, CDK7 homologues in model yeast predominantly regulate transcription [11–13]. Recently, we demonstrated that CDK7 from the pathogen *Cryptococcus neoformans* (*Cn*), forms a complex with Cyclin H and Mat1 that predominantly localizes to the nucleus [14]. Using the human CDK7 inhibitor SY-1365, a pyrimidine-based chemotherapeutic agent, we also showed that *Cn*CDK7 is like mammalian CDK7 in that it is involved in both transcriptional and cell cycle regulation [14].

The pyrazolo[1,5-a] pyrimidine – derived inhibitor, BS-181, is another promising chemotherapeutic agent that inhibits the growth of a range of cancer cell lines, including those of leukemic origin [15,16]. BS-181 displays antitumor effects in xenograft mouse models, attributed to suppression of Ser5 phosphorylation in the Rpb1 CTD and G1 cell cycle arrest [8]. Its anti-leukemic activity against human T-ALL Jurkat T cells has also been linked to G1 arrest through induction of an extrinsic apoptotic pathway involving elevated surface TRAIL/DR5 [15]. Given its potential as a cancer therapy, BS-181’s activity is of particular interest in patients with blood cancers, who are often immunocompromised by the cancer treatment and highly susceptible to opportunistic fungal infections [17,18].

Although classically associated with AIDS, infections caused by the World Health Organization (WHO) fungal priority pathogen *Cn* [19] are increasingly being reported in patients with hematological and solid organ malignancy [20]. Infection is typically acquired by inhalation of infectious particles, where cryptococcal lung nodules can be mistaken for tumors [21]. *Cn* frequently disseminates to the brain, causing meningoencephalitis that is fatal without treatment [22,23]. The sister species *Cryptococcus gattii* (*Cg*) follows a similar pathogenesis but has less predilection for brain infection [24]. Current treatment involves amphotericin B, a membrane-targeting antifungal with nephrotoxic side-effects, followed by azole antifungals which also impact membrane integrity [25–27]. However, azoles are fungistatic, and their extensive and often prolonged use has led to rising resistance [22,23].

The antifungal activity and mode of action of BS-181 have not been investigated. Here we demonstrate that BS-181 inhibits cryptococcal CDK7 and slows *Cn* progression through G_2_/M. Transcriptomic analysis revealed that despite having minimal effect on phosphorylation of RNAPII in *Cn*, BS-181 suppresses post-transcriptional processes such as splicing resulting in wide-spread intron retention, as well as translation and protein synthesis. BS-181 also showed the strongest antifungal effect *in vitro* and in animal models when combined with licensed, membrane-targeting antifungals.

## Results

### Cryptococcal CDK7 more closely resembles human CDK7, and BS-181 inhibits CnCDK7 enzyme activity

**Figure 1.**
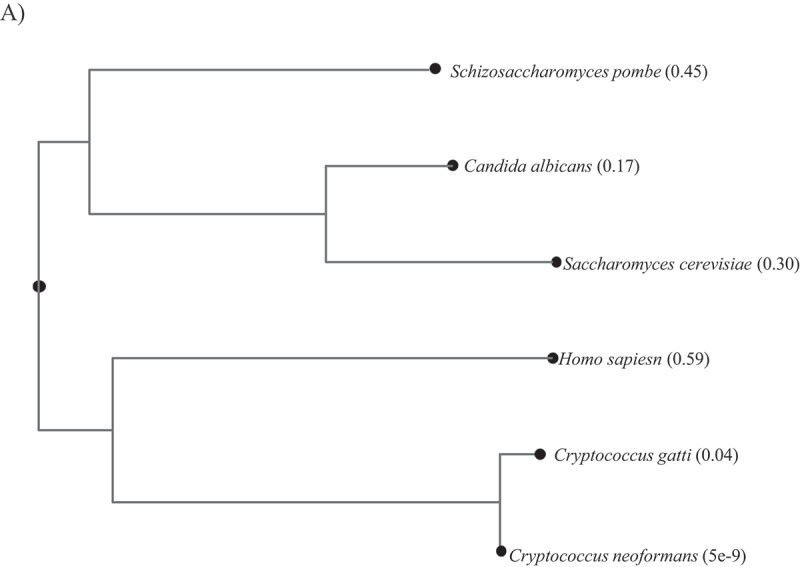
Phylogenetic/Guide trees obtained following amino acid sequence alignment of the CDK7 homologues from *Homo sapiens (hs)*, the ascomycetes yeasts *S. cerevisiae (sc), S. pombe (sp)* and *C. albicans (ca)*, and the basidiomycetes yeasts, *C. neoformans (Cn)* and *C. gatti (Cg).* The tree branches into two major clusters representing ascomycetes and basidiomycetes, with *Hs*CDK7 clustering with the basidiomycete homologues. *Cn*CDK7 (CNAG_06445), *Cg* (CNBG_5015), *Hs*CDK7 (NP_001790.1), *Sc*CDK7/*Sc*Kin28 (NP_010175.1), *Sp*CDK7/*Sp*Mcs6 (SPBC19F8.07) and *Ca*Kin28 (C1_06710W_A). Numbers represent pairwise evolutionary distances between the sequences. The tree was constructed using clustalW (https://www.genome.jp/tools-bin/clustalw) first by performing multiple sequence alignment followed by tree construction using fast program.

**Figure 2.**
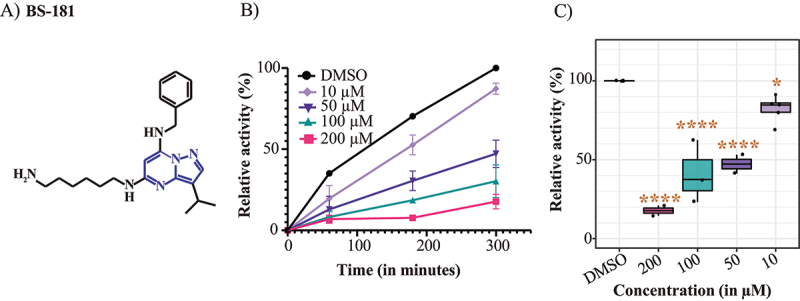
BS-181 inhibits *Cn*CDK7. (A) chemical structure of the pyrazolopyrimidine CDK7 inhibitor, BS-181. (B) In vitro kinase assays were performed over a 300 min time course in the absence (DMSO) and presence of the indicated concentrations of BS-181, using pulled-down CDK7, a CDK7 peptide substrate and kinase-Glo® reagent. ATP consumption (due to the phosphorylation of the peptide substrate by CDK7) was measured as a relative luminescence unit (RLU). The enzyme activities at each time point are plotted and expressed as “relative activity (%)” after normalization as described in the methods and represent the mean relative activity ± SEM (*n* = 2–3 independent experiments). In (C), the mean relative enzyme activity ± SEM (*n* = 2–3 independent experiments) at the 300 min (from B) is plotted and statistical analysis was performed using ordinary one-way ANOVA with Dunnett’s multiple comparisons test. Asterisks indicate P-values compared to the DMSO control. *p < 0.05, ****p < 0.0001.

Phylogenetic analysis shows that CDK7 from the basidiomycetous yeasts, *Cn* and *Cg*, cluster more closely with human CDK7 than do homologues from ascomycetes yeasts, including the priority pathogen *Candida albicans* (Figure 1). Based on this similarity, we hypothesized that the human CDK7 inhibitor, BS-181 (Figure 2(A)) also inhibits cryptococcal CDK7. To address this, we carried out an *in vitro* kinase assay using mNeonGreen-tagged *Cn*CDK7 that was pulled down from *Cn* lysates, and a CDK7-specific peptide substrate corresponding to the C-terminal domain (CTD) of Rbp1, the largest subunit of RNA Polymerase II (RNAPII). CDK7 phosphorylates Ser5 in the CTD of Rpb1 to initiate transcription. Creation of the *Cn*CDK7 tagged strain was described previously where it was shown that *Cn*CDK7 forms a complex with Cyclin H and Mat1 that predominantly localizes to the nucleus [14]. Dose-response curves were conducted, and the last time point was plotted against a no-inhibitor (DMSO) control to demonstrate that BS-181 suppressed the relative activity of *Cn*CDK7 in a dose-dependent manner (Figure 2(B-C)).

### BS-181 is more antifungal against Cn and Cg than against S. cerevisiae and C. albicans

**Figure 3.**
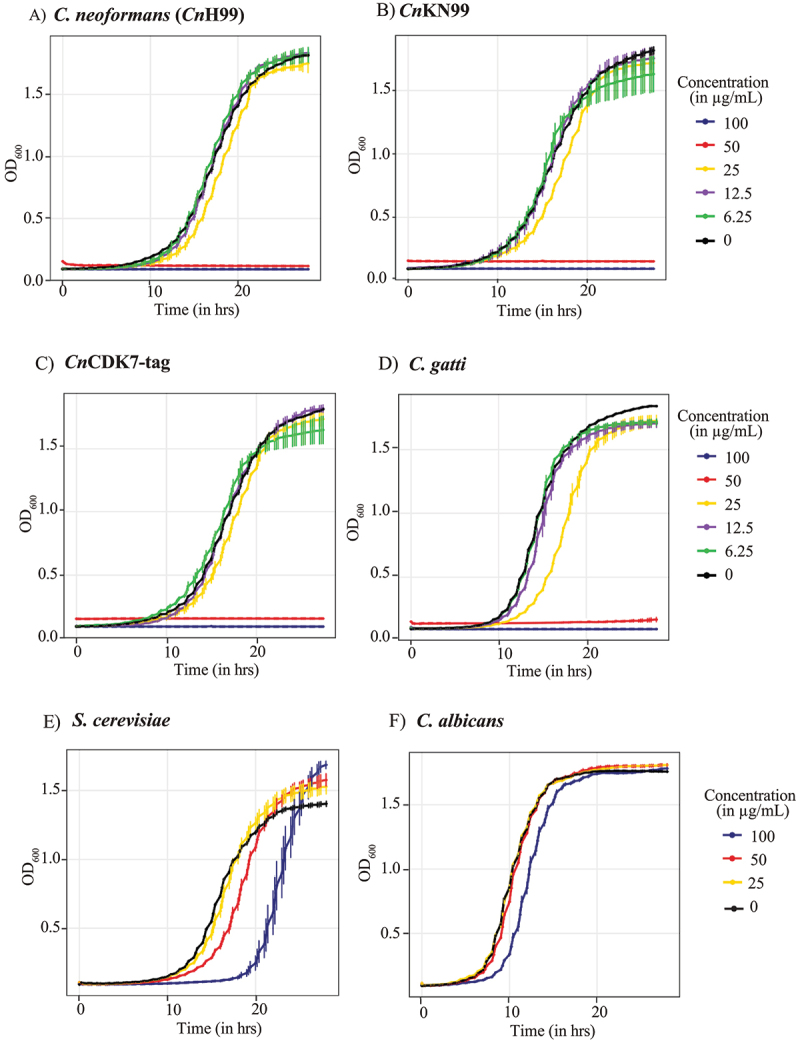
BS-181 elicits a higher inhibitory effect against *Cn* and *Cg* than *S. cerevisiae* and *C. albicans*. Overnight cultures of the indicated strains were used to seed fresh media to an OD_600_/mL = 0.01, and dose-response curves were generated over 28 h for *Cn* strain H99 (A), *Cn* strain KN99 (B), the CDK7-tagged strain (made in the KN99 background) (C), *C. gattii* (D), *S. cerevisiae* (E) and *C. albicans* (F). Growth in 96-well plates was determined by recording OD_600_ readings every 20 min using an Agilent Biotek log phase 600 plate reader. Growth inhibition was calculated as a percentage (%) relative to the no drug treatment control (0.5% DMSO). The experiment was performed with biological triplicates.

To test whether BS-181 inhibits fungal growth, a dose-response experiment was performed on *Cn* (strains H99, KN99 and KN99-CDK7-Tag), *Cg* (strain R265), *S. cerevisiae* (strain BY4741) and *C. albicans* (strain ATCC90028) with growth monitored spectrophotometrically over 28 h. The KN99 strain was tested because the CDK7 used for the enzyme assay was tagged in this strain [14]. The results demonstrate that BS-181 concentrations of ≥50 µg/mL, completely inhibited growth of all cryptococcal strains, consistent with a fungicidal effect (Figure 3(A-D)). Some growth inhibition was also observed at 25 µg/mL, especially for *Cg*. However, 50 µg/mL of BS-181 did not inhibit growth of *S. cerevisiae* nor *C. albicans* and 100 µg/mL only partially inhibited both strains (Figure 3(E) and (F)). These results are consistent with the more distant relationship of the CDK7 homologues of these two species to human CDK7, as compared to *Cn*CDK7 (Figure 1).

### BS-181 slows Cn progression through the G_2_/M phase of the cell cycle

**Figure 4.**
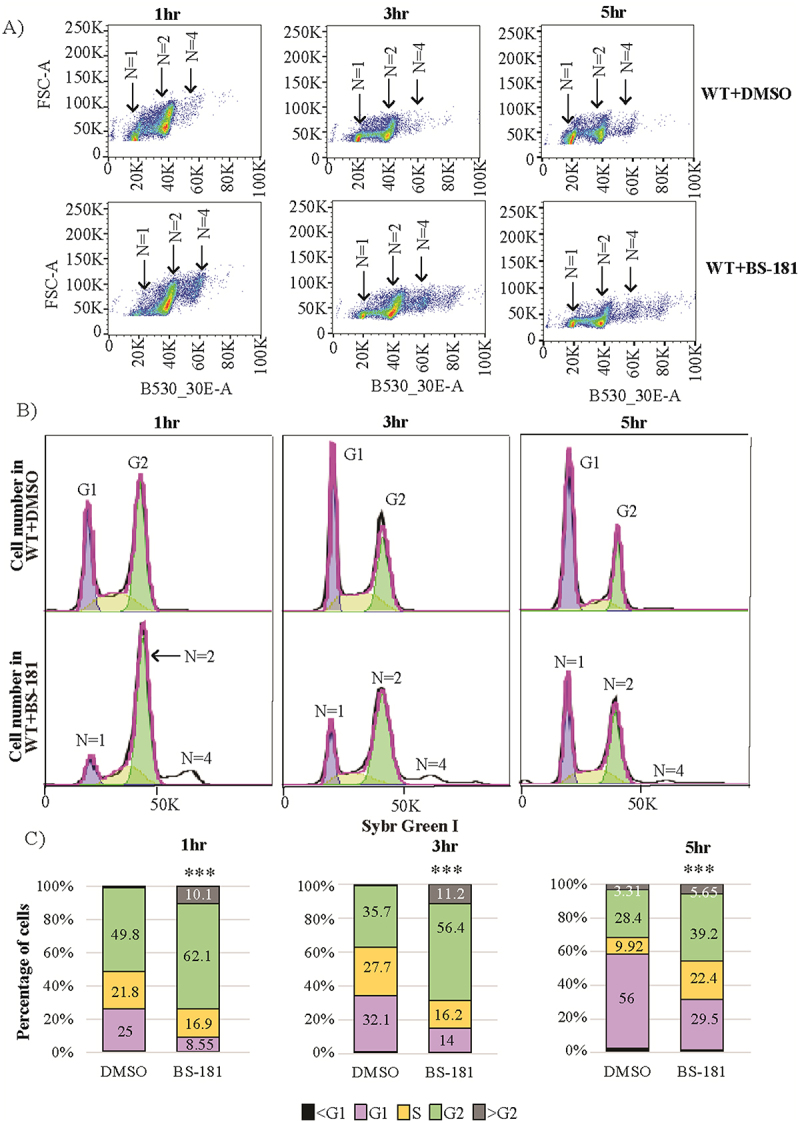
Effect of BS-181 on the *Cn* cell cycle. Treated and untreated cells were grown to log phase (see supplemental fig. S1) and fixed and stained with SYBR-green for analysis by flow cytometry. A plot of forward scatter height (FSC-H) versus forward scatter area (FSC-A) enabled gating on single (non-budding) cells (see supplemental fig. S1). (A) shows a plot of the fluorescence intensity of the gated single cells [FSC-A versus SYBR-channel (B530_30E-A)] where 3 distinct cell populations are observed (*N* = 1, *N* = 2 and *N* = 4), with the proportion of *N* = 4 cells being higher in drug-treated cells. (B) are the histograms generated from (A) showing a decrease and increase in the number of cells in G_1_ (*N* = 1) and G_2_ (*N* = 2), respectively, after 1, 3 and 5 h treatment with BS-181 as compared to untreated cells (0.1% DMSO). Drug treatment also led to an accumulation of cells with a DNA content greater than G_2_ (*N* = 4). Purple- and green-shaded peaks depict the G1 and G_2_ phases, respectively, and the yellow-shaded curves depict the S phase. (C) Graphic representation of the histograms indicating the percentage of cells in each phase. Only percentages greater than 1% are indicated. Asterisks indicate that the growth phase differences between BS-181 treated *Cn* and untreated *Cn* (DMSO) are statistically significant (*p* < 0.001, *n* = 2 biological replicates, chi-square test).

To further investigate the antifungal activity of BS-181, we tested its impact on the cell cycle by comparing the proportion of BS-181-treated *Cn* and untreated *Cn* in each cell cycle phase using flow cytometry (Figure 4). Both growth curves (Figure S1A) and cell imaging (Figure S1B) indicate that, prior to permeabilization and SYBR Green staining for flow cytometric analysis, both treated and untreated *Cn* cells were in log phase. This is consistent with healthy cells and confirms the use of a sublethal dose of BS-181. Staining with the vital nucleophilic dye DAPI, which exhibits restricted uptake in cells with intact membranes, was comparable between treated and untreated cells at 1 and 5 h and was predominantly confined to the cell periphery. Budding was most prevalent at 3 h, and although BS-181-treated cells exhibited increased DAPI signal at this time point, staining was diffuse and largely peripheral. In log phase, untreated *Cn* had a doubling time of ~120 min, in agreement with what others have found (132 min ±16) [28], and drug-treated cells grew slower than untreated cells over the 5 h treatment period used for the flow cytometry analysis. After gating on the single cell population which excluded budding cells (Figure S1C), a plot of forward scatter vs SYBR green fluorescence identified 3 distinct cell populations at all treatments times, corresponding to *N* = 1, *N* = 2 and *N* = 4 (Figure 4(A)). The histograms (Figure 4(B) and C) indicate that after 1 h of treatment with BS-181, the percentage of these single cells that were in the G_1_ phase (*N* = 1) was reduced to 8.6% from 25% for the DMSO-treated control. This coincided with an increased proportion of cells in G2 from 49.8% to 62% in DMSO and BS-181-treated cells respectively, and with DNA content greater than G2 (*N* = 4). A similar pattern was observed after 3 h and 5 h of treatment.

## Transcriptomics reveal BS-181 suppresses critical processes in Cn despite minimal effect on RNAPII phosphorylation

**Figure 5.**
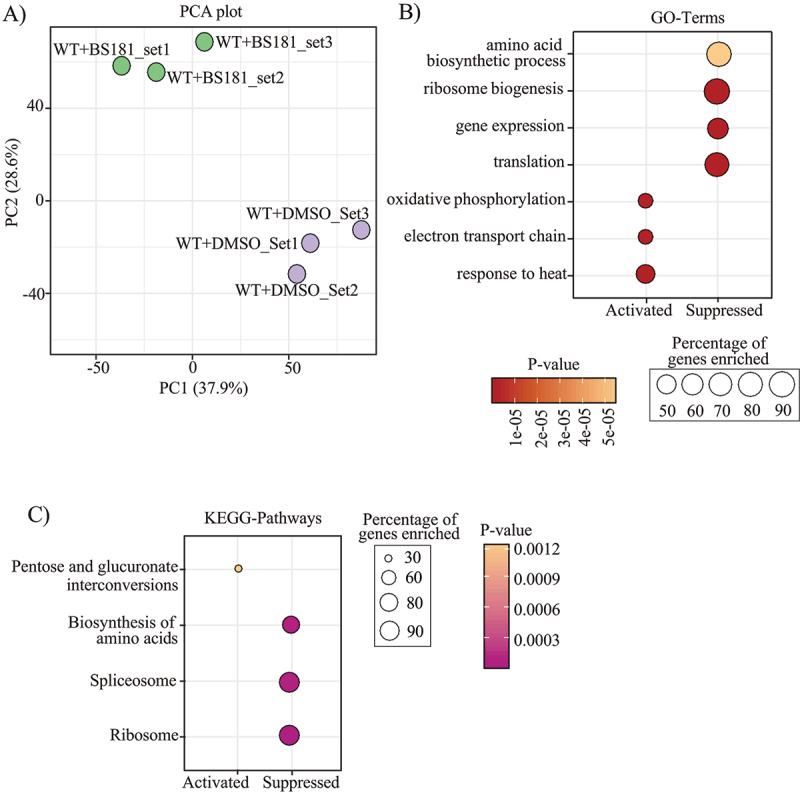
RNA-seq data analysis identifies functions that are suppressed and upregulated by BS-181-treatment. RNAseq was performed on DMSO (control) and 50 µg/mL BS-181-treated *Cn* following 1 h exposure (*n* = 3) as described in the methods. (A) A PCA plot shows that replicates correlate well, with each treatment group having a distinct cluster profile. Gene Set enrichment analysis (GSEA) using gene ontology (GO) terms (B) and KEGG pathways (C), identified similar processes and pathways that were suppressed (translation and splicing) and activated (heat stress response and mitochondria-associated energy generation) by BS-181 treatment. Table S1 lists all the genes used in GSEA.

Given that BS-181 inhibits phosphorylation of the CDK7 peptide substrate Y_1_S_2_P_3_T_4_S_5_P_6_S_7_ in the enzyme assay (Figure 2), we investigated the impact of BS-181 treatment of *Cn* on the phosphorylation of Ser5 and Ser2 using anti-Rpb1-Ser5p and anti-Rpb1-Ser2p Western blotting (Figure S2). Compared to treatment with DMSO, treatment with 50 or 100 μg/ml of BS-181 for 30 min (A) or 50 μg/ml BS-181 over longer times (B), caused a small reduction in phosphorylation of Rpb1 on Ser5 (Rpb1-Ser5p) and Ser2 (Rpb1-Ser2p) which, following normalization to the loading controls, was not statistically significant (Figure S2). Despite the minimal impact that BS-181 had on phosphorylation of RNAPII, RNAseq (Figure 5(A)) followed by Gene Set Enrichment Analysis (GSEA) using both Gene Ontology (GO) terms (Figure 5(B)) and KEGG pathways (Figure 5(C)), identified processes and pathways that were suppressed (translation and splicing) and activated (heat stress response and mitochondria-associated energy generation) by BS-181 treatment. The list of genes used in the GSEA analysis is shown in Table S1.

## BS-181 treatment leads to intron retention in Cn

**Figure 6.**
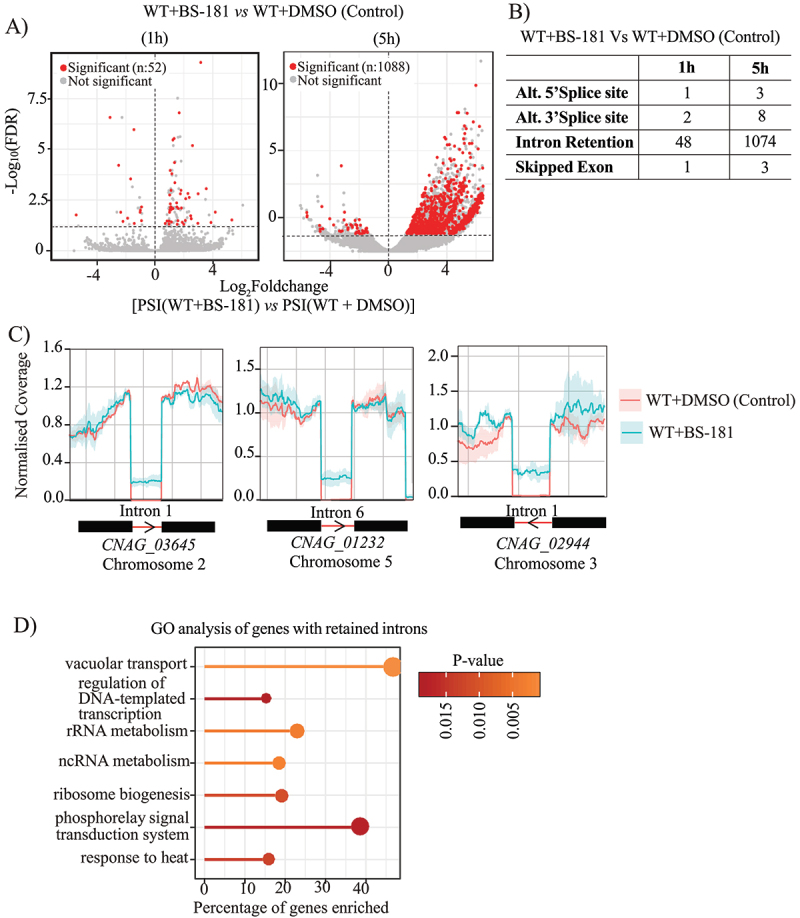
BS-181 treatment leads to intron retention in *Cn.* The RNA seq data (1 h and 5 h) was re-analyzed to quantify ASEs in 50 µg/mL BS-181 and DMSO (control) treated *Cn*. (A) A volcano plot of – log_10_FDR versus the Log_2_FC in PSI (treated vs. untreated) shows the distribution of all ASEs detected at 1 h and 5 h (gray dots) post-BS-181 treatment. Red dots represent the significant ASEs (FDR < 0.05 and ∆PSI > 5%) above the horizontal dotted line that indicates the FDR cutoff (-Log10FDR > 1.3). (B) Table showing that intron retention (IR) is the most prevalent ASE detected post BS-181 treatment. (C) Representative coverage plots of significant IR events in *CNAG_03645, CNAG_01232* and *CNAG_02944* post-treatment. These genes are positioned on the right-hand side of the vertical line in A and B. (D) GO analysis of the genes with retained introns (table S4) (n:1039, P-value < 0.05).

To further investigate the role of *Cn*CDK7 in co-transcriptional regulation, we reevaluated the RNA sequencing data from Figure 5 to compare the frequency of alternative splicing events (ASEs) in BS-181-treated and untreated *Cn*, using the R package *SpliceWiz* [29]. ASEs were quantified using the Percent Spliced-In (PSI) metric. Volcano plots of – log_10_false discovery rate (FDR) versus the Log_2_FC in PSI (treated *vs*. untreated) shows the distribution of all ASEs detected after BS-181 treatment for 1 h and 5 h (Figure 6(A), gray dots). Using a filtering threshold of ΔPSI ≥5% (treated – control) and a FDR < 0.05 (–log10[FDR] > 1.3), 52 (~0.2%) and 1088 (~4%) of all the ASEs detected at 1 h (Table S2) and 5 h (Table S3) treatment, respectively, were found to be significant (Figure 6(A), red dots). Among these significant ASEs, Intron Retention (IR), as opposed to Alternate 5’/3’ Splice Sites (A5SS/A3SS) and Skipped Exons (SE), was the predominant ASE at both time points (Figure 6(B)). This is consistent with IR being the most common ASE in *Cn* in response to stress [30]. Representative coverage plots of significant ASEs are shown in Figure 6(C), where BS-181 treatment led to the retention of introns in *CNAG_03645, CNAG_01232* and *CNAG_02944*. GO analysis of the genes with retained introns (n:1039, Table S4) identified several functions in common with the RNASeq analysis (Figure 5(B)) including translational regulation and a heat stress response. Transcriptional regulation, vacuolar transport and signaling were also identified (Figure 6(D)).

## The antifungal activity of BS-181 is enhanced in combination with licensed antifungals

The antifungal activity of BS-181 was also tested when combined with two classes of licensed membrane perturbing antifungals – azoles and polyenes – against both laboratory and clinical strains of *Cn* and *C. gattii* (*Cg*) using a standardized checkerboard susceptibility assay. Azoles, such as fluconazole, inhibit ergosterol biosynthesis, whereas polyenes, such as amphotericin B (AmB), bind directly to ergosterol disrupting fungal membrane integrity and function [25–27].

The MIC of BS-181 as a stand-alone agent, was similar to that obtained in the shaking broth cultures in Figure 2(A). However, in combination with AmB at concentrations 2–4-fold below its standalone MIC, the MIC of BS-181 was markedly reduced by ~90-fold (to 0.56 µg/mL) and ~30-fold (to 1.56 µg/mL) against the *Cn* (H99) and *Cg* (R265) laboratory strains, respectively (Table 1). The FICI values for the BS-181/AmB combinations against *Cn* and *Cg* were 0.31 and 0.52, respectively, reflecting synergy and additivity. The antifungal activity of BS-181/AmB against *Cn* and *Cg* clinical isolates was also tested. In nearly all isolates, BS-181 potency once again increased considerably when combined with AmB, with low to sub µM concentrations (0.4–1.56 µg/mL) required to achieve a predominantly additive effect (FICI = 0.5–0.53; Table 2). This compared to a FICI of 0.75 and 0.56 (additive) for the AmB/flucytosine combination against *Cn* and *Cg*, respectively. AmB/flucytosine is standard therapy for initial treatment of cryptococcal infections [22,23]. Hence, combining BS-181 with AmB results in an at least similar, if not more efficacious, effect *in vitro* as compared to standard of care therapy.

**Table 1.** Antifungal activity of BS-181 against C. neoformans and C. gattii laboratory strains alone and in combination with amphotericin B (AmB) or fluconazole (Flu).

Drug(s)	For *Cn* (H99)			For *Cg* (R265)		
	Interaction	FICI	MIC(s) (µg/mL)	Interaction	FICI	MIC(s) (µg/mL)
AmB			0.25–0.5			0.25–0.5
Flu			4			4
BS-181			50–100			50–100
AmB + BS-181	Synergy	0.31	AmB: 0.125, BS-181: 0.56	Additive	0.52	AmB: 0.25, BS-181: 1.56
Flu + BS-181	Additive	0.63	Flu: 0.5, BS-181: 50	Synergy	0.38	Flu: 1, BS-181: 25

MIC: minimal inhibitory concentration alone or in combination therapy (µg/mL); FICI: fractional inhibitory concentration index where FICI ≤ 0.5 is synergistic, 0.5< FICI ≤1 is additive, 1.0 < FICI ≤ 4.0 is indifferent and >4.0 is antagonistic. The FICI for the AmB/flucytosine combination against Cn and Cg was 0.75 and 0.56 (additive) against Cn and Cg, respectively (not shown in Table).

**Table 2.** Antifungal activity of BS-181 against C. neoformans and C. gattii clinical isolates in combination with amphotericin B (AmB) or fluconazole (Flu).

	BS-181/AmB(MIC = µg/mL)	BS-181/fluconazole (Flu)(MIC = µg/mL)	AmB/flucytosine(MIC = µg/mL)
*C. gattii* (lung 6457) Accession #WMD 2021.001	Additive	Synergy	Additive
	FICI = 0.52	FICI = 0.38	FICI = 1
	MIC AmB = 0.25	MIC Flu = 0.25	
	MIC BS-181 = 1.56	MIC BS-181 = 25	
*C. gattii* (blood) Accession #WMD 2022.002	Synergy	Synergy	Additive
	FICI = 0.50	FICI = 0.50	FICI = 0.53
	MIC AmB = 0.25	MIC Flu = 0.25	
	MIC BS-181 = 1.56	MIC BS-181 = 25	
*C. gattii* (bronchial wash) accession #WMD 2022.003	Additive	Synergy	Additive
	FICI = 0.52	FICI = 0.50	FICI = 0.63
	MIC AmB = 0.25	MIC Flu = 0.5	
	MIC BS-181 = 0.78	MIC BS-181 = 25	
*C. gattii* (CSF) accession # WMD 2022.004	Additive	Synergy	Additive
	FICI = 0.51	FICI = 0.50	FICI = 0.63
	MIC AmB = 0.25	MIC Flu = 0.5	
	MIC BS-181 = 0.78	MIC BS-181 = 25	
*C. gattii* (lung-3251) accession #WMD 2022.005	Synergy	Synergy	Additive
	FICI = 0.50	FICI = 0.50	FICI = 0.56
	MIC AmB = 0.25	MIC Flu = 0.5	
	MIC BS-181 = 0.39	MIC BS-181 = 25	
*C. neoformans* (blood) accession #WMD 2022.006	Additive	Synergy	Additive
	FICI = 0.63	FICI = 0.50	FICI = 0.75
	MIC AmB = 0.25	MIC Flu = 1.0	
	MIC BS-181 = 12.5	MIC BS-181 = 25	
*C. neoformans* (CSF) accession #WMD 2022.007	Additive	Additive	Additive
	FICI = 0.53	FICI = 0.63	FICI = 0.75
	MIC AmB = 0.25	MIC Flu = 0.13	
	MIC BS-181 = 1.56	MIC BS-181 = 25	
*C. neoformans* (leg nodule) accession # WMD 2022.008	Additive	Synergy	Additive
	FICI = 0.52	FICI = 0.50	FICI = 0.75
	MIC AmB = 0.25	MIC Flu = 0.5	
	MIC BS-181 = 1.56	MIC BS-181 = 25	
*C. neoformans* (bronchial wash) accession # WMD 2022.009	Indifferent	Synergy	Additive
	FICI = 2	FICI = 0.50	FICI = 2
	MIC AmB = 0.5	MIC Flu = 0.5	
	MIC BS-181 = 100	MIC BS-181 = 25	

MIC: minimal inhibitory concentration in combination therapy (µg/mL); FICI: fractional inhibitory concentration index where FICI ≤ 0.5 is synergistic, 0.5< FICI ≤1 is additive, 1.0 < FICI ≤ 4.0 is indifferent and >4.0 is antagonistic. FICIs for AmB/flucytosine are included as a comparison.

In combination with fluconazole at concentrations 4–8-fold below its standalone MIC, the MIC of BS-181 was also reduced to achieve additivity or synergy against the *Cn* (H99) and *Cg* (R265) laboratory strains (Table 1). However, this 2–4-fold reduction in the BS-181 concentration in the presence of fluconazole, was less than that observed in the presence of AmB. The efficacy of BS-181 against *Cn* and *Cg* clinical isolates also improved, mostly by 4-fold, in the presence of fluconazole concentrations that were 4–8-fold below its standalone MIC, to mostly achieve synergy (Table 2). Our results are consistent with an increased ability of BS-181 to penetrate *Cn* cells in the presence of AmB and fluconazole.

## BS-181 + AmB is more protective than AmB against Cn infection in an insect model

**Figure 7.**
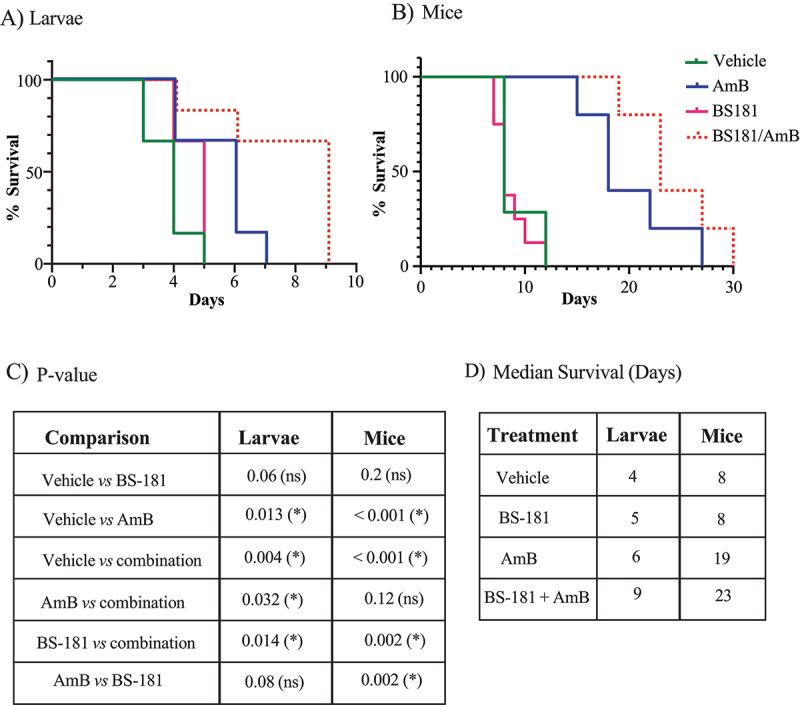
BS-181 combination therapy prolongs survival of *Cn*-infected insect larvae (A) and mice (B). In (A) larvae (*n* = 7–9 per group) were injected with 7.5 × 10^5 *Cn* followed by injection with 0.5% DMSO (vehicle), BS-181 (100 mg/kg), AmB (5 mg/kg) or BS-181 (100 mg/kg) + AmB (5 mg/kg). Survival was monitored daily for up to 9 days. The results are a representative experiment of 2 biological replicates where similar results were obtained. In (B), mice (5–6 per group) were infected via the retroorbital plexus with 1 × 10^5 *Cn* (Day 0). On Day 1, 2 and 3 post-infection, mice were injected intraperitoneally with vehicle (7.5% DMSO, 3.75% tween-20, in 50 mM HCl in saline), BS-181 (10 mg/kg), AmB (2 mg/kg) or BS-181 (10 mg/kg) + AmB (2 mg/kg). Mice were euthanized when they reached their clinical end point (see methods). (C) Table depicting statistics (p-value) determined using a log rank (mantel-Cox) test. ns: not significant. (D) Median survival (in days) of insect larvae and mice post treatment with drugs or vehicle.

Next, we tested the efficacy of BS-181 alone and in combination with AmB, against *Cn* infection in a *Galleria mellonella* model (Figure 7(A)). We focused on AmB combination therapy (rather than fluconazole) because AmB reduced the effective BS-181 concentration to micromolar to sub micromolar levels. The BS-181/AmB combination therapy prolonged larval survival relative to AmB treatment (9- versus 6-days median survival respectively; *p* = 0.032) and BS-181 treatment (9- versus 5-days median survival, respectively; *p* = 0.014). Relative to the no treatment group, AmB treatment increased median survival of larvae by two days (*p* = 0.013), while the one-day increase in median survival observed for BS-181-treatment compared to no treatment was not quite significant (*p* = 0.06).

We also tested the efficacy of BS-181/AmB combination therapy in an intravenous mouse model of infection (Figure 7(B)). Unlike the larvae experiment, which required a single high dose of BS-181 to avoid repeated injections that would cause injury and premature death, mice received three consecutive daily doses of each drug, alone or in combination, via the peritoneal cavity. In line with ethical requirements, mice were euthanized upon reaching defined clinical endpoints (see Methods). Consistent with mouse tumor model studies [8], at 10 mg per kg of BS-181, no weight loss was observed before infection symptoms developed, indicating a lack of toxicity. BS-181/AmB treatment prolonged survival by 4 days compared with the AmB only group, although this trend was not statistically significant. The p-values and median survival for both the galleria and mouse experiments are summarized in Figures 7(C) and 7(D), respectively.

## Discussion

Our results show that cryptococcal CDK7 shares greater amino acid similarity with human CDK7, than with ascomycete homologues. This correlates with BS-181 inhibiting *Cn*CDK7 activity, and more effectively suppressing growth of cryptococcal species than *S. cerevisiae* or *C. albicans*.

## Comparison with cancer cells

Although BS-181 affects cryptococcal function differently from what has been reported in human cancer cell lines, the overall outcome is similar – cell cycle arrest and inhibition of translation, primarily through post-transcriptional processing. In cancer cells, BS-181 treatment caused G_1_ accumulation [16], whereas in *Cn* we observed G_2_/M arrest. These differences may reflect the fact that in *Cn*, a single CDK7-regulated CDK (CDK1/CDC2) controls the cell cycle [31–33], while in mammalian cells multiple CDK7-regulated CDKs (CDK1/2/4/6) are involved. Alternatively, since BS-181 is derived from roscovitine, an ATP-competitive inhibitor of CDK1, CDK2, CDK5, and CDK7, it could also act on *Cn*CDK1. However, BS-181 is 40-fold more potent against human CDK7 than CDK2 supporting CDK7 as the primary target. Importantly, G_2_/M arrest was also observed when *Cn* was treated with SY-1365, a pyrimidine-based chemotherapeutic that inhibits *Cn*CDK7 more potently than BS-181 [14]. This supports a CDK7-specific effect on the cryptococcal cell cycle. In contrast, *S. cerevisiae* Kin28 does not regulate the cell cycle [11].

## Comparison with SY-1365

Transcriptomic analysis revealed some mechanistic differences between BS-181 and what was previously reported for SY-1365 [14]. In *Cn*, BS-181 had little effect on RNAPII phosphorylation and thus limited impact on transcription initiation and elongation. Instead, it suppressed post-transcriptional processes, causing widespread intron retention. Many of the affected genes were involved in transcription and translation, although additional processes such as vacuolar transport and signaling were also impacted. In contrast, SY-1365 did inhibit RNAPII Ser2/Ser5 phosphorylation, suppressing both transcription initiation and elongation and consequently translation and, interestingly, had less of an impact on splicing [14].

Several factors may explain these differences: the transient nature of RNAPII phosphorylation, the reversible inhibition by BS-181 versus covalent inhibition by SY-1365 as observed for human CDK7 [10], differences in bioavailability (SY-1365 being more potent and bioavailable), and target specificity. Regarding specificity, CDK7 exists as a free CAK complex or is bound to TFIIH. BS-181 May preferentially inhibit free *Cn*CAK while SY-1365 targets both free and TFIIH-associated *Cn*CAK. This would explain why both drugs cause G_2_/M arrest in *Cn*, yet SY-1365 has a stronger impact on transcription initiation and elongation [14]. Moreover, as free CAK in mammalian cells can phosphorylate transcriptional CDKs such as CDK9 [3], which plays a role in both transcription elongation and splicing [34], BS-181 may alter transcription indirectly in *Cn* by driving intron retention. Intron retention has been reported as a stress response in *Cn* that reduces gene expression without increasing proteome diversity [30]. Thus BS-181-induced splicing defects may suppress gene expression and translation in *Cn* through a mechanism distinct from SY-1365. Future studies using kinase assays with free versus TFIIH-associated CAK and defined peptide substrates will be critical to assess these mechanisms.

## Other cellular impacts

The anti-leukemic activity of BS-181 has been attributed to G1 arrest and induction of apoptosis through mitochondrial dysfunction [16]. In contrast, our RNAseq data indicate that in *Cn*, BS-181 upregulated mitochondrial activity, which may reflect a CDK7-specific or nonspecific stress response. BS-181 also induced a heat stress response.

## In vivo antifungal potential

On its own, BS-181 has a high MIC, potentially because it cannot effectively penetrate the fungal cell wall and/or the fungal membrane where the predominant sterol is ergosterol rather than cholesterol. Given that the MIC of BS-181 was reduced predominantly to low µg/ml or sub µg/ml levels in the presence of AmB, the synergy with AmB is likely to be due to the weakened plasma membrane caused by AmB binding or soaking up ergosterol [25–27] and thus facilitating uptake of BS-181. Consistent with its anti-tumor activity in mouse xenograft models [16], BS-181 also showed antifungal efficacy in combination therapy. BS-181 plus AmB provided greater protection against *Cn* infection in a wax moth model than either drug alone, and extended survival in mice compared to AmB alone, though not significant with the current dosing regimen. Importantly, we showed that BS-181 was not toxic in infected mice when administered at 10 mg/kg for 3 consecutive days. This is based on the observation that infected mice administered BS-181 alone or in combination with AmB, did not lose weight faster than non-drug-treated infected mice. Our study agrees with that of others who assessed the antitumor activity of BS-181 in a mouse model, where 10–20 mg/kg administered daily for 14 days was also well tolerated based on the absence of weight loss [8]. This suggests that higher and/or more frequent BS-181 dosing, or reduced AmB dosing, could enhance therapeutic benefit in the mouse infection model.

In summary, we demonstrate that BS-181 inhibits cryptococcal CDK7, blocks progression through G_2_/M, and induces widespread intron retention that may suppress gene expression and translation. These findings establish BS-181 as an antifungal with a distinct mechanism from SY-1365. They also highlight the potential of CDK7 inhibitors as dual-use agents – chemotherapeutics and antifungals – particularly valuable for blood cancer patients at high risk of opportunistic infections, including cryptococcosis.

## Methods

### Strains, Media and Inhibitors

The *Cn* strains used were H99, KN99 and KN99-CDK7-Tag. Strain H99 was used for growth and susceptibility testing, cell cycle analysis, transcriptomics and animal studies. KN99 [35], which is derived from H99, was used as the source of CDK7 (from KN99-CDK7-Tag) in the enzyme assay. *Cg* (strain R265), *Saccharomyces cerevisiae* (strain BY4741) and *Candida albicans* (strain ATCC90028) were also used in the growth assay. *Cn* and *Cg* clinical isolates were sourced from the Clinical Mycology Laboratory at Westmead Hospital. Unless indicated otherwise, the growth media was YPD. The CDK7 inhibitor, BS-181 (≥99% purity Cat. No.: HY-13266, 5 mg), flucytosine (Cat. No Hy-B0139-5 g) and Amphotericin B (Cat. No.: HY-B0221, 100 mg) were purchased from MedChemExpress through Focus Bioscience Pty Ltd, Queensland, Australia. Fluconazole was purchased from Sigma-Aldrich (Cat No. F8929-100 mg). Stock solutions were prepared in 100% DMSO (Sigma, Cat. No D4540 100 ml).

### Assessing the antifungal properties of BS-181

The antifungal properties of BS-181 were assessed using an Agilent Biotek log phase 600 plate reader for 28 h as described in [14], with 0.5% DMSO serving as the vehicle (no drug) control. An R-package, growkar (https://sethiyap.github.io/growkar/) was implemented to plot cell growth as Time *vs* OD_600_. Custom scripts used for plot generation are available at:https://github.com/sethiyap/CnCDK7_analysis/blob/main/Paper_BS181.md The experiments were performed in biological triplicates.

### Antifungal susceptibility testing

Antifungal susceptibility testing was assessed using the European Committee on Antimicrobial Susceptibility Testing (EUCAST) method as previously described [14], https://www.eucast.org/astoffungi/methodsinantifungalsusceptibilitytesting/susceptibility_testing_of_yeasts. The minimum inhibitory concentration (MIC) is defined as the lowest drug concentration to inhibit growth by 100%. The fractional inhibitory concentration index (FICI) is the antifungal effect of combination therapy where FICI ≤0.5 is synergistic, 0.5 < FICI ≤1 is additive, 1.0 < FICI ≤4.0 is indifferent and > 4.0 is antagonistic [36].

### Animal studies

*Galleria mellonella* infection: Studies were performed as previously described [37] *G. mellonella* larvae (*n* = 7–9 per group weighing ~250 mg) were injected with 7.5 × 10^5^ *Cn* (10 µL), incubated at 37°C for 2 h and injected with 0.5% DMSO (vehicle), BS-181 (100 mg/kg), AmB (5 mg/kg) or BS-181 (100 mg/kg) + AmB (5 mg/kg). Survival was monitored daily for up to 9 days post-infection. *Mouse infection model*: Mouse infection studies were performed as described [38,39] using female BALB/c mice weighing ~20 g obtained from Australian BioResources (Moss Vale New South Wales). Access to food (autoclavable rat and mouse chow by Specialty Feeds) and water was unrestricted. The light-dark cycle was 12 h. Mice were acclimatized for 1 week before experiments commenced. Briefly, mice (5–6 per group) were infected via the retroorbital plexus with 150 µL of saline containing 1 × 10^5^ *Cn* (Day 0). On Day 1, 2 and 3 post-infection, mice were injected intraperitoneally with 200 µL of vehicle (7.5% DMSO, 3.75% Tween-20, in 50 mM HCl in saline), BS-181 (10 mg/kg), AmB (2 mg/kg) or BS-181 (10 mg/kg) + AmB (2 mg/kg). Mice were weighed daily for up to 30 days and euthanized with CO_2_ when they had lost 15% of their pre-infection weight or before if they showed debilitating clinical symptoms of infection including meningitis and lethargy. Statistics: For both the larvae and mouse experiments, median survival and p-values for the various pair-wise comparisons, determined using a log rank (Mantel-Cox) test, are indicated in Figure 7. Ethical approval Statement: All mouse experiments performed in this study were undertaken according to protocol 4361.02.22 which was approved by the Western Sydney Local Health District animal ethics committee.

### Flow cytometry and cell cycle analysis

Flow cytometry and cell cycle analysis on strain *Cn*H99 treated with wither 50 µg/mL BS-181 or 0.1% DMSO (control) for 1, 3 or 5 h was performed as described previously [14]. Growth curves (*n* = 2 biological replicates) were performed by recording OD_600_ readings to confirm that cells were in log phase. Cell morphology and health were also assessed at these time points using Delta Vision fluorescence microscopy. Sample processing for flow cytometric analysis was also performed as described in [14]. Briefly, cell pellets were fixed in 75% ethanol, treated with 0.5 mg/mL RNase A (Thermo Fisher Scientific AM2286 1 ml) and 1x Sybr Green I (Thermo Fisher Scientific S7563 500 µl), washed, sonicated in 50 mM Tris-HCl pH 7.5 and run on a BD FACS Canto II system. Flow data was analyzed using the FlowJo cell cycle analysis function. Statistical significance of growth phase differences between BS-181 treated *Cn* and untreated *Cn* (DMSO) were assessed using a Chi-Square test.

### Western blotting to detect phosphorylated Rpb1

*Cn*H99 cultures were grown overnight, diluted to an OD_600_ of 4 in fresh medium, and incubated for 2 h at 30 °C. Cells were then treated with BS-181 (50 or 100 µg/mL) or an equivalent volume of DMSO (0 µg/mL BS-181) as a control for 30 min to 3 h. At each time point, cells corresponding to an OD_600_ of 20 were harvested, snap-frozen in liquid nitrogen, and stored at − 80 °C. Proteins were extracted with TRIzol reagent (Thermo Fisher Scientific; Cat. No. 15,596,026) and separated by SDS-PAGE as previously described [39,40]. Phosphorylation of the RNA polymerase II Rpb1 subunit at Ser5 and Ser2 was detected using a monoclonal anti-phospho-Rpb1 Ser5 antibody (Abcam; clone 3E8, Cat. No. ab252852) and a polyclonal anti-phospho-POLR2A Ser2 antibody (Proteintech; Cat. No. 30,888–1-AP) as described in [14].

### RNA extraction and library preparation

*Cn*H99 was grown overnight in YPD medium at 30 °C with shaking (250 rpm). Cultures were diluted to an OD_600_ of 4 in biological triplicate and incubated for 2 h. Cultures were then treated with BS-181 (50 µg/mL) or an equivalent volume of DMSO for 1 h or 5 h. Cells were harvested, snap-frozen, and stored at − 80 °C. Total RNA was extracted using TRIzol (Thermo Fisher Scientific; Cat. No. 15,596,026), purified with the Qiagen RNeasy MinElute Cleanup Kit (Cat. No. 74,104) and assessed for integrity using an Agilent TapeStation. Samples with an RNA integrity (RIN) score > 8 were used for library preparation as described previously [14,37].

### RNA sequencing analysis

RNA-seq data analysis was performed as previously described [14,37,41]. Raw FASTQ reads (SRA accession: PRJNA1293871) were quality-checked using FastQC software [42], adaptor-trimmed using the fastQ pre-processor tool, fastp (v: 0.19.6) [43] and aligned to the *C. neoformans* H99 genome (FungiDB-59) using the STAR alignment tool (v:2.7.8a) [44]. Gene-level read counts were obtained using the R-package Rsubread [45] and normalized, followed by differential expression analysis of BS-181–treated versus DMSO-treated samples using the R-package DESeq2 (v:1.42.1) [46] after filtering low-abundance genes (read counts < 10). Functional enrichment was assessed by Gene Set Enrichment Analysis (GSEA) using the R-package ClusterProfiler [47], and exploratory analyses was performed via the FungiExpresZ portal [48]. Visualizations were generated using in-house R scripts (https://github.com/sethiyap/CnCDK7_analysis).

### Splicing analysis on RNAseq data

The same RNAseq data was used for splicing analysis with raw fastq reads mapped to the *Cn* reference genome (Cryptococcus_neoformans_var_grubii_h99_gca_000149245.CNA3.dna.genome.fa) obtained from the Ensembl database using STAR aligner (v:2.7.8a) [44]. The R-package SpliceWiz was implemented to determine Alternate Splicing Events (ASEs) [29] as previously described [14]. Custom scripts for splicing analysis and visualization are available at:https://github.com/sethiyap/SpliceFungi

### Testing CDK7 inhibitors with an *in* *vitro* kinase assay using pulled down CDK7

mNeonGreen-tagged CDK7 was pulled down (as a CAK complex) from lysates of the CDK7-tagged strain created in the KN99 background, using ChromoTek mNeonGreen-Trap Agarose beads (Cat. No. nta, from Proteintech sourced through United Bioresearch, Sydney Australia) as previously described [14,49]. The pulled down CAK complex was then used to conduct kinase assays using 10 µM ATP and 250 µM of the peptide substrate, CDK7/9tide, in the presence of 5% DMSO or various concentrations of BS-181 as previously described [14]. Kinase-Glo® reagent (Cat. No. V6713, Promega) was then used to stop the reactions and to measure the remaining ATP as a relative luminescence unit (RLU). Luminescence was measured using a SpectraMax iD5 instrument and the RLU values were used to calculate the Relative Activity (%) of CDK7 as described in [14].

## Guidelines statement

This study has been conducted in accordance with the ARRIVE guidelines.

## Supplementary Material

SupplementaryData.docx

## Data Availability

All raw RNASeq fastq files for this study are publicly available on the NCBI Sequence Read Archive (PRJNA1293871) https://www.ncbi.nlm.nih.gov/sra/PRJNA1293871. The data-processing workflow is described in the Methods section and is available on GitHub (https://github.com/sethiyap/CnCDK7_analysis; https://github.com/sethiyap/SpliceFungi). The raw data underlying the figures, and the supplementary tables, are available on Zenodo at https://doi.org/10.5281/zenodo.18025772 [50].
