# Supplementary material for: The cyclin dependent kinase (CDK)7 inhibitor BS-181 inhibits pathogenic Cryptococcus species, causing G2/M arrest and a splicing defect
Source: Virulence. 2026 Feb 17;17(1):2629100. doi: 10.1080/21505594.2026.2629100 (PMC12915825; doi:10.1080/21505594.2026.2629100)
Supplement: SupplementaryData.docx [file KVIR_A_2629100_SM8470.docx]

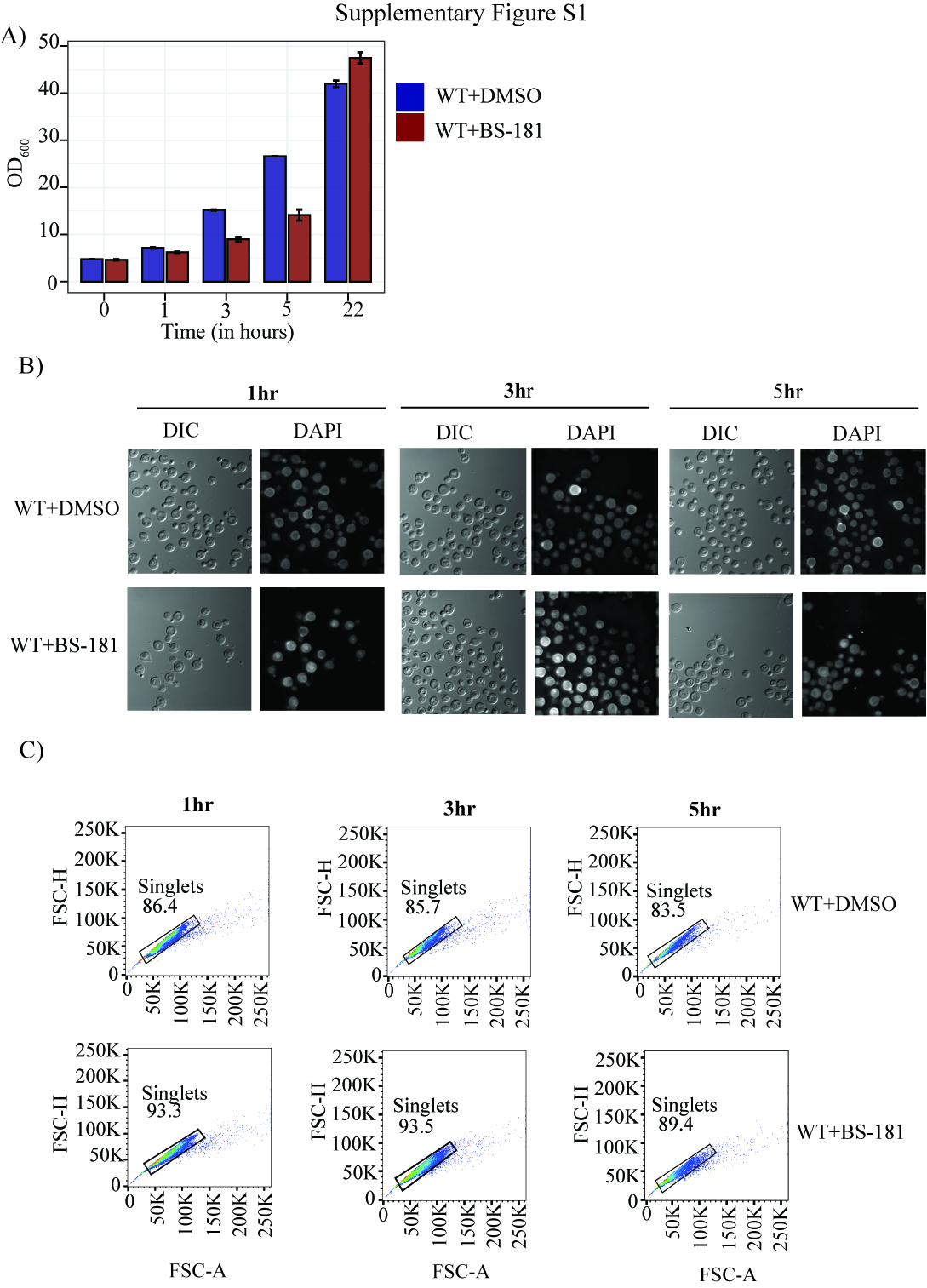


**Figure S1 Growth conditions and gating strategy to select single *Cn* cells for cell cycle analysis by flow cytometry. (A)** BS-181-treated (50 μg/mL) and untreated cells were grown for 22 h in biological duplicate, with growth assessed spectrophotometrically (OD600) at the indicated times. **(B)** Prior to fixation and staining for flow cytometry, *Cn* morphology at 1 h, 3 h and 5 h was assessed by fluorescence microscopy. In both treated and untreated samples, budding cells are observed (which were excluded from the flow analysis) and DAPI staining is mostly confined to the cell periphery, confirming that cells are healthy and intact. **(C)** A plot of forward scatter height (FSC-H) versus forward scatter area (FSC-A) and the gating strategy used to exclude budding cells.


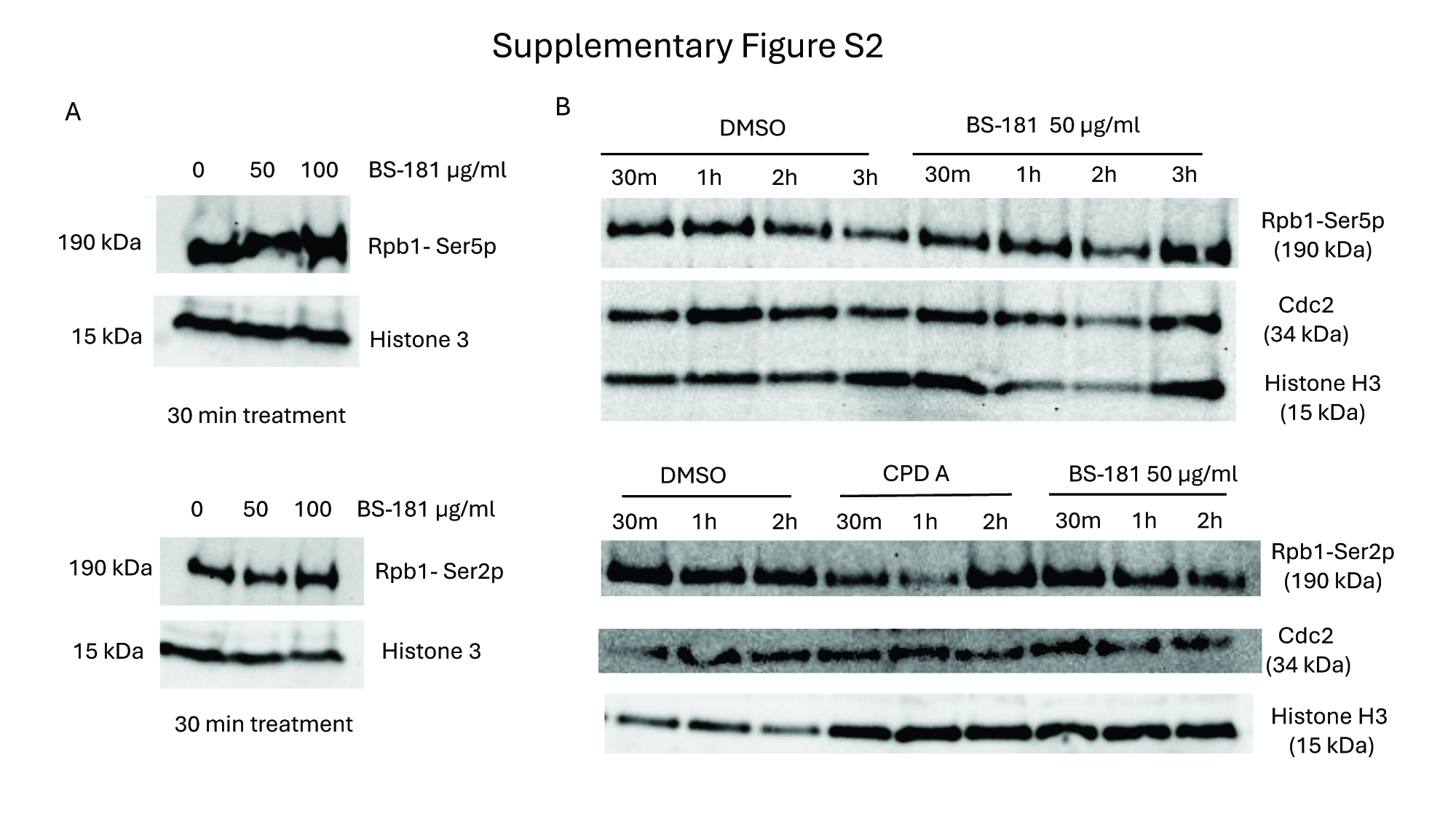


**Figure S2**. **BS-181 treatment of** ***Cn* does not inhibit phosphorylation of Ser5 and Ser2 in the CTD of the Rpb1 subunit of RNAPII.** Representative Western blots showing that, compared to treatment with DMSO, treatment with 50 or 100 µg/ml of BS-181 for 30 min **(A)** or 50 µg/ml BS-181 over longer times **(B)** does not significantly inhibit phosphorylation of Rpb1 on Ser5 (Rpb1-Ser5p) or Ser2 (Rpb1-Ser2p) when normalized to Histone H3 or Cdc2, which were used as loading controls. Specifically, 30 min treatment with 50 µg/mL BS-181, caused a 32% reduction in mean phosphorylation on Ser5 ± STD compared to untreated (normalized to 1.0) but was not statistically significant (0.68±0.3245, n=4, p=0.1419, T-test), and a 14% reduction in phosphorylation on Ser2 ± STD compared to untreated (normalized to 1.0) but was not statistically significant (0.86±0.49, n=5, p = 0.43, T-test). Compound (CPD) A is unrelated to this study but was included to show the whole blot.

**Table S1**: List of genes used in GSEA of WT+BS-181 *vs* WT+DMSO (Control) (1h) RNASeq data

**Table S2**: Alternate Splicing Events in WT+BS-181 *vs* WT+DMSO (Control) post 1h of treatment

**Table S3**: Alternate Splicing Events in WT+BS-181 *vs* WT+DMSO (Control) post 5h of treatment

**Table S4**: List of genes with retained IR in WT+BS-181 *vs* WT+DMSO (Control) post 5h of treatment used for GO analysis
